# Neuralgia of the Jaw Bones

**Published:** 1871-01

**Authors:** 


### NEURALGIA OF THE JAW BONES.

Prof. Gross says: "There is a form of neuralgia of the jaw bones which, as far as my information extends, has not hitherto been described. Its seat is in the remnants of the alveolar process of edenlulous persons, or in the structure, and in the overlying gum, and is met with chiefly, if not exclusively, in elderly subjects. It is more common in the upper than in the lower jaw. The part affected is usually very small, often not exceeding a few lines in extent. The soft tissues around do not seem to suffer, at least not in the same degree; on the contrary, the morbid action is generally limited to the osseous structure. In rare instances there may possibly be some involvement of the gum, which is nearly always exceedingly hard and dense, grating more or less under the knife, and adhering with extraordinary firmness to the atrophied alveolar process beneath. The pain is generally paroxysmal, coming on in fits and starts, very much as in ordinary neuralgia, the slightest causes being sufficient to provoke it, as talking, mastication, the contact of hot or cold fluids, deglutition, or mental excitement. Sometimes it is momentary, coming and going with the rapidity of lightning; occasionally it lasts for hours together; and cases occur, although they are rare, in which it continues, with but little mitigation, for an indefinite period. The pain varies in character; thus it may be sharp and darting, dull, heavy, aching, boring, or gnawing. Pressure generally relieves rather than aggravates it. Now and then, when it is uncommonly severe, there may be more or less spasm of the muscles of the face, but this is rare. The pathology of the affection seems to be compression of the minute nerves distributed through the wasted alveolar process, dependent upon the encroachments of osseous matter upon the walls of the canals in which they are naturally inclosed. The disease usually comes on gradually, and proceeds from bad to worse until, in many cases, the suffering is rendered nearly intolerable. The only effectual remedy is excision of the affected alveolar process. No particular attention need be bestowed upon the after treatment. A mild course of chalybeate tonics may be required when the patient is anæmic, or affected with flatulence and indigestion."—*Amer. Jour. Med. Science.*

